# Supplementary material for: Taxonomic analysis of metagenomic data with kASA
Source: Nucleic Acids Res. 2021 Mar 30;49(12):e68. doi: 10.1093/nar/gkab200 (PMC8266618; doi:10.1093/nar/gkab200)
Supplement: gkab200_Supplemental_Files [file gkab200_supplemental_files.zip › Suppl_D.zip!/OPAL_comparison_new/gold_standard/family.pdf]

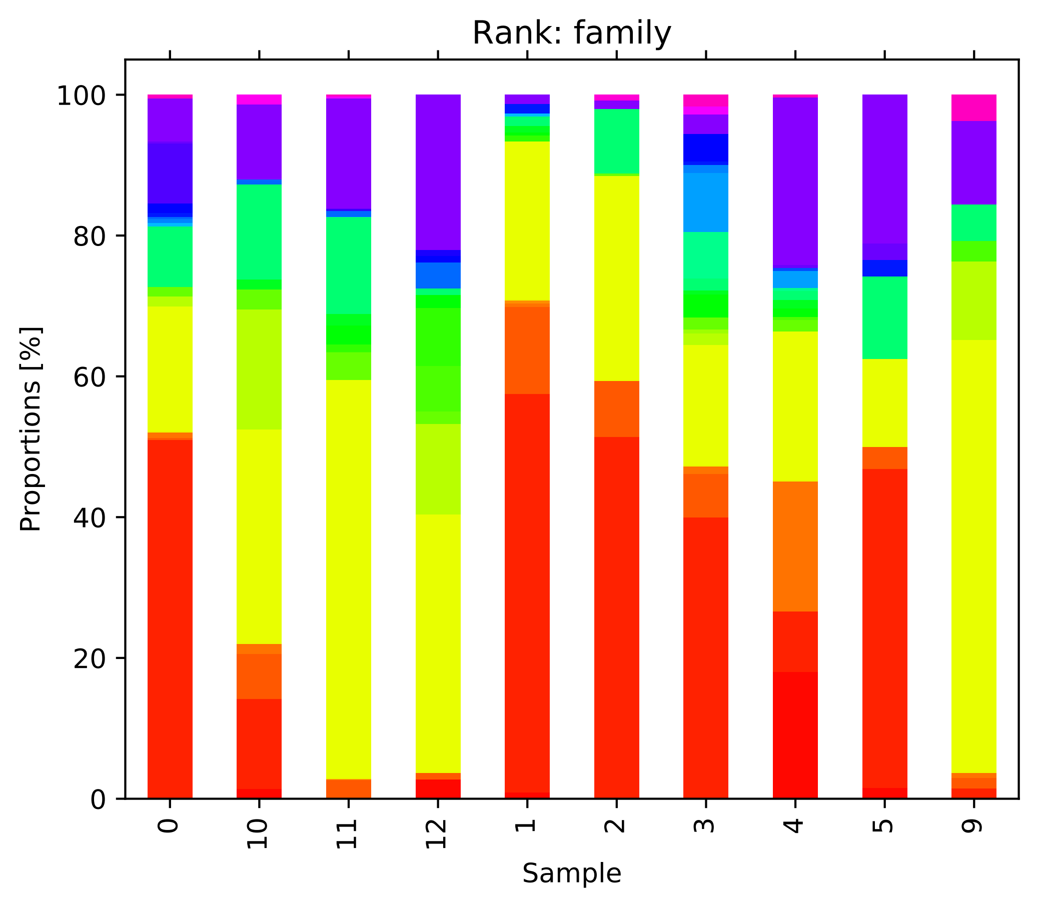
Rank: family
Proportions [%]
0
20
40
60
80
100
0
10
11
12
1
2
3
4
5
9
Sample
